# Case report: A rare case of acute hemolysis in advanced rectal cancer after XELOX and nivolumab treatment: analysis of drug-dependent antibodies

**DOI:** 10.3389/fmed.2023.1167759

**Published:** 2023-05-25

**Authors:** Dengke Zhao, Qiao Song, Chunyan Wu, Shuye Wang

**Affiliations:** ^1^Harbin Medical University, Harbin, China; ^2^Department of Medicine, Qingdao University, Qingdao, China; ^3^Department of Hematology, Affiliated Hospital, Qingdao University, Qingdao, China; ^4^Department of Hematology, The First Affiliated Hospital, Harbin Medical University, Harbin, China

**Keywords:** drug-related hemolytic anemia, colorectal cancer, oxaliplatin, nivolumab, treatment

## Abstract

**Objective:**

To investigate the mechanism, *in vitro* differential test and clinical significance of hemolytic anemia after receiving oxaliplatin and nivolumab treatment.

**Methods:**

We encountered a male patient with stage IV rectal cancer who experienced acute hemolysis during the ninth cycle of treatment with XELOX combined with nivolumab and cetuximab. The patient's blood samples were collected and tested for the presence of oxaliplatin or nivolumab antibodies on red blood cells.

**Results:**

Direct antiglobulin testing of red blood cells incubated with oxaliplatin was strongly positive, whereas cells incubated with nivolumab were negative, which suggested that oxaliplatin was responsible for the hemolysis. After short-term highdose glucocorticoid treatment, human normal immunoglobulin infusion, and other symptomatic treatments, the patient's condition rapidly improved, and he continued to receive nivolumab treatment without further hemolysis.

**Conclusion:**

Attention should be paid to the possibility of acute hemolysis when using oxaliplatin and nivolumab, and it is important to recognize and manage this adverse event early. We detected oxaliplatin-related antibodies on the surface of red blood cells *in vitro*, which provided evidence for the following treatments.

## Introduction

Oxaliplatin is a key component of chemotherapy used as a first-line treatment for advanced colorectal cancer with relatively few side effects (1). Oxaliplatin immune-induced syndrome (OIIS) includes severe acute hemolytic anemia and/or thrombocytopenia and symptoms such as fever, sudden back pain, and hematuria (2–4). Programmed death cell protein 1 (PD-1) inhibitors can block tumor immune tolerance, among them, nivolumab has been used to treat mismatch repair deficient/microsatellite instability-high (dMMR/MSI-H) metastatic colorectal cancer (5). Nivolumab can cause side effects such as hypothyroidism, diarrhea, decreased appetite, colitis, pneumonitis, hepatic function abnormal, sepsis, acute kidney injury, uveitis (6). Nivolumab also has several immune-related adverse reactions including rash, pruritus, and rarely, immune hemolytic anemia, which could be life-threatening (7–9). Drug-induced hemolytic anemia may be either “warm” or “cold” depending on the temperature at which the autoantibodies become active. Warm autoimmue hemolytic anemia (AIHA) is caused mainly by warm-reactive IgG-mediated extravascular hemolysis, Cold AIHA results from IgM activation of classic complement pathway and the autoantibodies are active at temperatures 4°C (10). Some cases that both types of autoantibodies may exist simultaneously are labeled as “mixed” AIHA (10). Increasing reports of oxaliplatin-induced or nivolumab-induced hemolytic anemia have been published, however, cases of acute hemolysis after receiving oxaliplatin and nivolumab treatment were rarely reported. Only few of the published cases did drug-dependent red blood cell (RBC) antibodies tests *in vitro* which confirmed that hemolysis was directly related to the drug. We encountered a case of acute hemolysis in a patient with stage IV rectal cancer during treatment with XELOX combined with nivolumab and cetuximab. Oxaliplatin-related antibodies were detected on the surface of erythrocytes *in vitro*, which provided evidence for the following treatments.

## Case report

**Figure 1:**
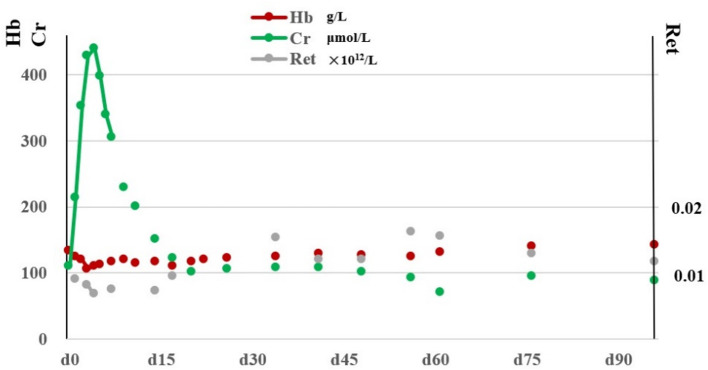
Changes of hemoglobin, creatinine and reticulocyte count after hemolysis. ^*^Hb, hemoglobin; Cr, creatinine levels; Ret, reticulocyte.

A 47-year-old Chinese male patient initially presented with a malignant rectal tumor (pTxN2bM1b, stage IV B). PET-CT revealed liver and bone metastasis. From November 2020 to March 2021, he completed eight cycles of XELOX combined with nivolumab and cetuximab. The cumulative dose of oxaliplatin was 716 mg/m^2^ (1,440 mg), and that of nivolumab was 955 mg/m^2^ (1,920 mg). Two hours after the end of oxaliplatin administration during the ninth treatment cycle, the patient complained of sudden lower back pain and fatigue, and his urine was strongly brown. Laboratory analysis revealed hematuria (urine RBC count, 90.6/μL) and liver dysfunction (serum glutamic oxaloacetic transaminase, 142U/L; total bilirubin, 136.5 μmol/L). The direct antiglobulin test (DAT) was positive, and the indirect antiglobulin test (IAT) was negative. Considering drug-related hemolysis, follow-up chemotherapy drug was immediately stopped. He was managed with 1 mg/kg/day methylprednisolone from the first day after hemolysis was suspected (D1) at decreasing doses over 1 month, and human immunoglobulin (0.4 g/kg/day) was administered from D3 to D7. He received renal protective therapy (D3 to D21) because of the creatinine elevation. The symptoms of hemolysis resolved on D4. Laboratory examination showed decreased serum creatinine levels and increased hemoglobin levels. DAT was negative on D26. Oxaliplatin was discontinued, the patient resumed treatment with capecitabine, cetuximab, and nivolumab. There was no hemolysis syndrome, and the patient's hemoglobin and creatinine levels normalized (Figure 1).

We obtained peripheral blood samples from the patient to test for drug-related hemolysis. The concentration of oxaliplatin injection [Qilu Pharmaceutical (Hainan) Co., Ltd, Haikou, China] was 2 mg/mL, Nivolumab (Bristol Myers Squibb Holdings Pharma, Ltd, New York, United States) was prepared at a concentration of 5 mg/mL (11). Mixtures of patient sera and 5% oxaliplatin-treated RBC suspension were incubated at 37°C for 1 h, centrifuged, washed three times in 0.9% saline, assessed for hemolysis/agglutination via visual assessment, and Coombs test by microcolumn agglutination method. As a control, sera from healthy AB donor instead of patient sera, and type O RBC suspension instead of oxaliplatin-treated RBC suspension. The experiment was repeated for nivolumab in the same manner. Patient sera incubated with oxaliplatin-treated RBC suspension group was positive on DAT, and the other results were negative.

## Discussion

Our patient was treated with oxaliplatin and nivolumab, both of which could cause hemolysis. To further clarify the drug causing hemolysis, we detected drug-dependent RBC antibodies *in vitro*. DAT using patient sera and oxaliplatin-treated red blood cells (RBCs) was positive, indicating that anti-IgG/C3 antibodies were present on the surface of RBCs. Meanwhile, IAT was negative, indicating that there were no anti-IgG/C3 antibodies in the patient's serum. DAT using RBCs incubated with nivolumab was negative, suggesting the absence of nivolumab-dependent antibodies on the RBC membrane. The patient continued to receive nivolumab after the resolution of hemolysis, and there was no further evidence of acute hemolysis, indicating that the hemolysis was caused by oxaliplatin.

Oxaliplatin is the third generation platinum antineoplastic drug, which inhibits the replication and transcription of DNA in tumor cells, thus inducing cell death. Combined with 5-fluorouracil derivatives and vascular endothelial growth factor in the treatment of advanced colorectal cancer, it can effectively improve patient survival and reduce disease recurrence (1, 12). Increasing reports of oxaliplatin-induced hemolytic anemia have been published, and some cases were accompanied by thrombocytopenia and neutropenia. It is characterized by fever, lower back pain, hematuria, abnormal liver function and renal function (2–4). Timely detection and treatment of these symptoms will help to prevent further deterioration of OIIS. The mechanism of oxaliplatin-induced hemocytopenia may include immune system activation (immune complex, non-immunologic drug adsorption, and true autoimmune), oxaliplatin pharmacokinetic, and genetic background predisposition, all of these may have played a role during the process (4, 11, 13–16). We consider that the antibody was already induced by previous oxaliplatin exposure. With repeated drug exposure, the immune response was enhanced until the antibody reached the threshold level, leading to hemolysis (17).

Several cases of acute renal injury after treatment with oxaliplatin have been reported, and these events were believed to be caused by the direct damage of renal tubules or the accumulation of hemolytic products in the renal tubules (18–20). In our case, the increased creatinine level appeared after hemolysis and improved rapidly after short-term high-dose glucocorticoid and immunoglobulin infusion. It is speculated that drug treatment can reduce the concentration of autoantibodies, resolve the cause of renal tubular necrosis, and gradually normalize renal function while hemolysis is alleviated. This clinical process supported the finding that renal insufficiency was caused by acute hemolysis.

Of the 61 patients reported, 27 (44.26%) received repeated oxaliplatin treatment. The median number of cycles before the occurrence of OIIS was four, which was significantly earlier than observed in patients who received other regimens (2). Vyskocil et al. (20) reported that they did Coombs test using patient's sera 6 months/12 months after the hemolysis with oxaliplatin-treated RBCs, the results showed long-term antibodies on the surface of RBCs, and platinum drug cross-reaction. Therefore, oxaliplatin treatment is no longer recommended for patients with OIIS.

Nivolumab blocks the binding of PD-1 and programmed death-ligand 1 (PDL-1) to tumor cells and stimulates T cells to target tumor cells. This effect of relieving T cell inhibition may be uncontrolled, resulting in autoimmune adverse reactions, including rash, pruritus, hemolytic anemia (21), immune hemocytopenia (7, 8, 22, 23). The mechanism of nivolumab-induced hemocytopenia is not clear but is likely related to reaction of T cells and drug-induced autoantibodies (24). DAT using RBCs incubated with nivolumab was negative, suggesting the absence of nivolumab-dependent antibodies on the RBC membrane. Thus, the patient continued to receive nivolumab, and there was no further evidence of acute hemolysis. Anemia usually occurs in the early stage of treatment. In the course of subsequent treatment, although RBC antibodies may already exist, the patient can still tolerate nivolumab (7).

## Conclusion

It is crucial to identify symptoms and signs of hemolysis and signs promptly as possible while receiving nivolumab and oxaliplatin anticancer treatment. The drug causing to hemolysis should be identified to provide evidence for follow-up drug selection.

## Data availability statement

The original contributions presented in the study are included in the article/supplementary material, further inquiries can be directed to the corresponding author.

## Ethics statement

Written informed consent was obtained from the individual(s) for the publication of any potentially identifiable images or data included in this article.

## Author contributions

DZ, QS, and CW were responsible for collecting data, sorting out data, and writing the article. SW was responsible for guiding the writing and participating in the revision of the article. All authors read and approved the final manuscript.
